# Differential immediate and long-term effects of nitrogen input on denitrification N_2_O/(N_2_O+N_2_) ratio along a 0‒5.2 m soil profile

**DOI:** 10.1371/journal.pone.0276891

**Published:** 2022-10-31

**Authors:** Haijing Yuan, Xinhua He, Jiafa Luo, Chunsheng Hu, Xiaoxin Li, Stuart Lindsey

**Affiliations:** 1 Key Laboratory of Soil Ecology, Center for Agricultural Resources Research, Institute of Genetics and Developmental Biology, Chinese Academy of Science, Shijiazhuang, China; 2 Center of Excellence for Soil Biology, College of Resources and Environment, Southwest University, Chongqing, China; 3 Land and Environment, AgResearch, Hamilton, New Zealand; Chinese Academy of Sciences, CHINA

## Abstract

High nitrogen (N) input to soil can cause higher nitrous oxide (N_2_O) emissions, that is, a higher N_2_O/(N_2_O+N_2_) ratio, through an inhibition of N_2_O reductase activity and/or a decrease in soil pH. We assumed that there were two mechanisms for the effects of N input on N_2_O emissions, immediate and long-term effect. The immediate effect (field applied fertilizer N) can be eliminated by decreasing the N input, but not the long-term effect (soil accumulated N caused by long–term fertilization). Therefore, it is important to separate these effects to mitigate N_2_O emissions. To this end, soil samples along a 0‒5.2 m profile were collected from a long-term N fertilization experiment field with two N application rates, that is, 600 kg N ha^-1^ year^-1^ (N600) and no fertilizer N input (N0). External N addition was conducted for each subsample in the laboratory incubation study to produce two additional treatments, which were denoted as N600+N and N0+N treatments. The results showed that the combined immediate and long-term effects led to an increase in the N_2_O/(N_2_O+N_2_) ratio by 6.8%. Approximately 32.6% and 67.4% of increase could be explained by the immediate and long-term effects of N input, respectively. Meanwhile, the long-term effects were significantly positively correlated to soil organic carbon (SOC). These results indicate that excessive N fertilizer input to the soil can lead to increased N_2_O emissions if the soil has a high SOC content. The long-term effect of N input on the N_2_O/(N_2_O+N_2_) ratio should be considered when predicting soil N_2_O emissions under global environmental change scenarios.

## Introduction

The N_2_O/(N_2_O+N_2_) ratio reflects the proportion of N_2_O accounting for the total main gaseous N emissions by denitrification, and its decrease is beneficial for the mitigation of soil N_2_O emissions in a certain extent. High N fertilizer input could cause higher N_2_O emissions, that is, a higher N_2_O/(N_2_O+N_2_) ratio. Increase in this ratio could result from the inhibition of soil N_2_O reductase activity and/or a decrease in soil pH [1]. We assumed that there should be considered the immediate and long-term effects of N input on N_2_O emissions. The immediate effect could be eliminated through a decrease in soil nitrate (NO_3_^-^) content, but it is more difficult to eliminate the long-term effects [2]. Numerous studies have investigated the enhancing effect of N input on soil denitrification and N_2_O emission [3–5], and the effect of N input on the end product composition of denitrification [6, 7]. However, these studies did not quantify the relative contributions of the immediate and long-term effects of N input on the N_2_O/(N_2_O+N_2_) ratio.

A separation of the immediate and long-term effects could provide a better understanding of the concurrent effect of continuous N input on soil N_2_O emissions and mitigation. In the present study, we collected soil samples along a 0‒5.2 m profile from a long-term N fertilization experimental field and conducted an external N addition experiment in a laboratory incubation study. The main aims of this study were to: 1) quantify the immediate and long-term effects of high N addition on the N_2_O/(N_2_O+N_2_) emission ratio and 2) analyze the correlation between soil properties and the N_2_O/(N_2_O+N_2_) ratio.

## Materials and methods

### Experimental site and soil sampling

The experimental site was located at the Luancheng Agro-Ecosystem Experimental Station (37.90°N, 114.67°E; elevation, 50 m) of the Chinese Academy of Sciences, Hebei, China. A long-term N fertilization experiment was established in 1998 with a winter wheat (*Triticum aestivum*) and summer maize (*Zea mays*) double cropping system. The experiment has four N fertilizer treatments each with three replicates: CK (0 kg N ha^−1^ year^-1^), low nitrogen (200 kg N ha^−1^ year^-1^), medium nitrogen (400 kg N ha^−1^ year^-1^) and high nitrogen (600 kg N ha^−1^ year^-1^). Two selected N treatments (0 and 600 kg N ha^−1^ year^-1^) were examined in this study, which are denoted as N0 and N600, respectively. Undisturbed soil columns (43 mm in diameter) of 0‒5.2 m depth (13 soil layers of 0.4 m) were collected in triplicates using a Geoprobe drilling rig (Geoprobe^®^54DT, USA) from the N0 and N600 treatments in October 2012. The collected composite soils within each layer were mixed and divided into two subsamples: one for soil physicochemical parameter analyses and one for measuring soil denitrification. Fresh soils were stored in poly vinyl chloride bags at 4°C before analysis. Details of other soil properties and specific field management have been previously described by Yuan *et al*. [8].

### Soil properties determination

Soil moisture was determined gravimetrically by oven drying samples to a constant weight at 105±0.5°C. Soil particle composition was determined using a laser particle size analyzer (Malvern Mastersizer 3000, UK). Soil pH was measured in a suspension of 1:5 soil to water using a pH meter. Soil NO_3_^-^ was extracted using a 1 M KCl solution in a soil/solution ratio of 1:5 (w/v) and determined using dual-wavelength ultraviolet spectrophotometry (Shimadzu UV2450, Japan) [9]. The total soil organic carbon (SOC) was determined using the dichromate oxidation method [10]. The soil parameters were analyzed in duplicate because of the limited soil volume in each layer.

### Laboratory incubation experiment

The laboratory experiment had three triplicate treatments using the composite soils: (I) soil sample N0 without N addition (N0), (II) soil sample N0 with N addition (N0+N) (III) soil sample N600 with N addition (N600+N). Previous studies have shown that dissolved organic carbon (DOC) content differs significantly within different soil depths and N fertilization rates [11]. Therefore, DOC was adjusted to the same level to eliminate the potential effects of DOC on the N_2_O/(N_2_+N_2_O) ratio. The N_2_O/(N_2_+N_2_O) ratio differences between N0+N and N0 were assumed to be the immediate effects, and the N_2_O ratio differences between N600+N and N0+N were considered as combined immediate and long-term effects. The procedure of a N0+N or N600+N was briefly described as follows: an equivalent of 10.0 g field-moist soil was agitated by a magnetic bar after adding 15 ml of 10 mM KNO_3_ and glucose mixture in a 120 ml serum flask. The flask was then capped with an air-tight butyl rubber stopper and aluminum crimp seal, and evacuated (0.1 kPa) and filled with high-purity helium gas (99.999%, 120 kPa) five times. The pressure of the headspace was adjusted to 101.3 kPa after the final helium filling. All flasks were anaerobic incubated (with helium filling) in a thermostatic water bath at 25°C. N_2_O and N_2_ measurements were started after a 1 hour incubation to establish equilibrium between the soil and headspace. The headspace gas in the flasks was sampled and analyzed for N_2_O and N_2_ concentrations using a robotized sampling and analysis system according to Molstad et al. [12]. The analysis for N0 sample followed the same procedure as the N0+N and N600+N treatments, with addition of no external N. The N_2_O and N_2_ emission rates were calculated by linearly regressing the headspace N_2_O and N_2_ concentrations with incubation time. The data of the initial couple of days were not used to calculate the N_2_O emission rate because N addition had obvious priming effects on N_2_O emission during this period.

### Data processing and statistical analysis

The figures were plotted using OriginPro9.0 (^©^Origin Lab Corporation) software. Analysis of variance (ANOVA) was performed to determine the difference in the N_2_O/(N_2_O+N_2_) ratio between the different N addition treatments using SPSS for Windows (version 16.0, SPSS Inc., Chicago). Duncan’s *post-hoc* test was used to assess the significant effects of the different N treatments on the N_2_O/(N_2_O+N_2_) ratio. Pearson’s correlations were analyzed between soil properties and immediate and long-term effects. Statistical significance was set at *p* < 0.05, unless otherwise stated.

## Results and discussion

**Fig 1:**
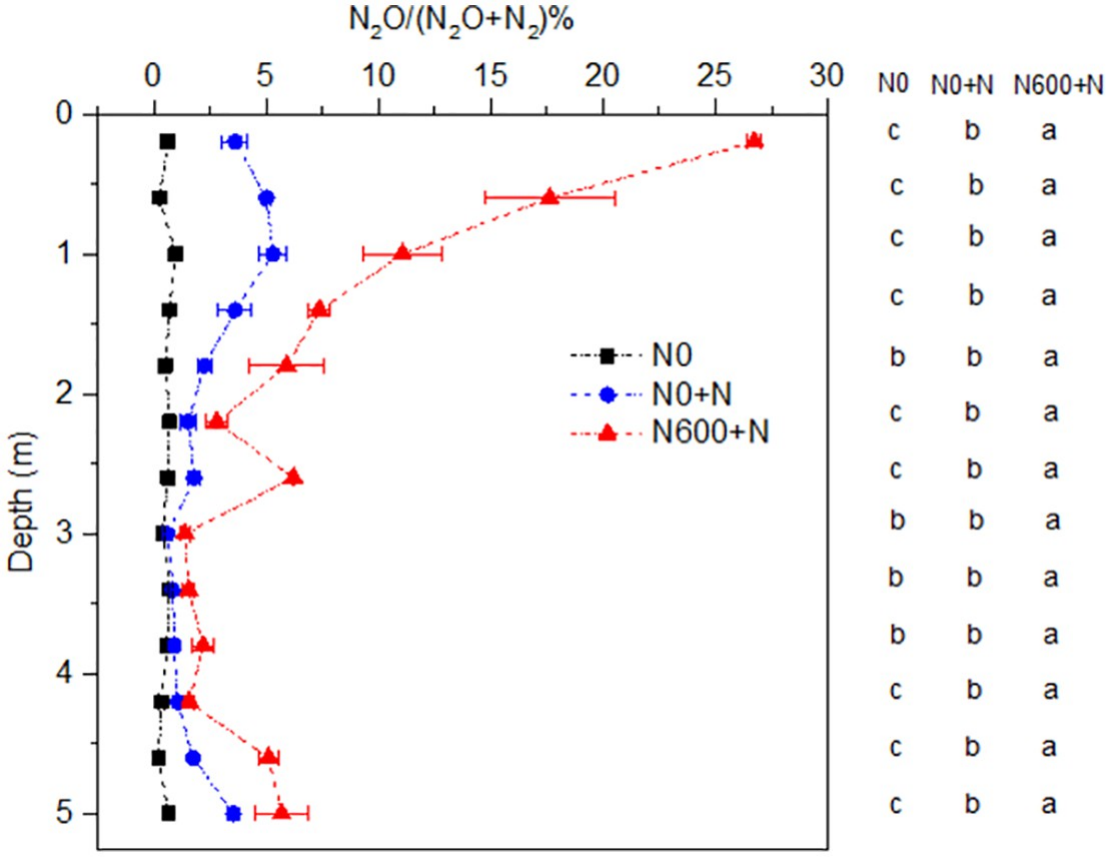
Soil N_2_O/(N_2_O+N_2_) emission ratio in different depths across the 0‒5.2 m soil profile in the N0, N0+N and N600+N treatments. N0 and N0+N represent laboratory anaerobic incubation using the soil N0 and N0+N, respectively. N600+N is the same as the N0+N treatment except for using the soil N600. N0 and N600 represent fertilizer N input rates of 0 and 600 kg N ha^-1^ year^-1^, respectively. Bars represent standard deviations of the means (n = 3). Different letters indicate significant difference at *p* < 0.05 between different treatments.

**Fig 2:**
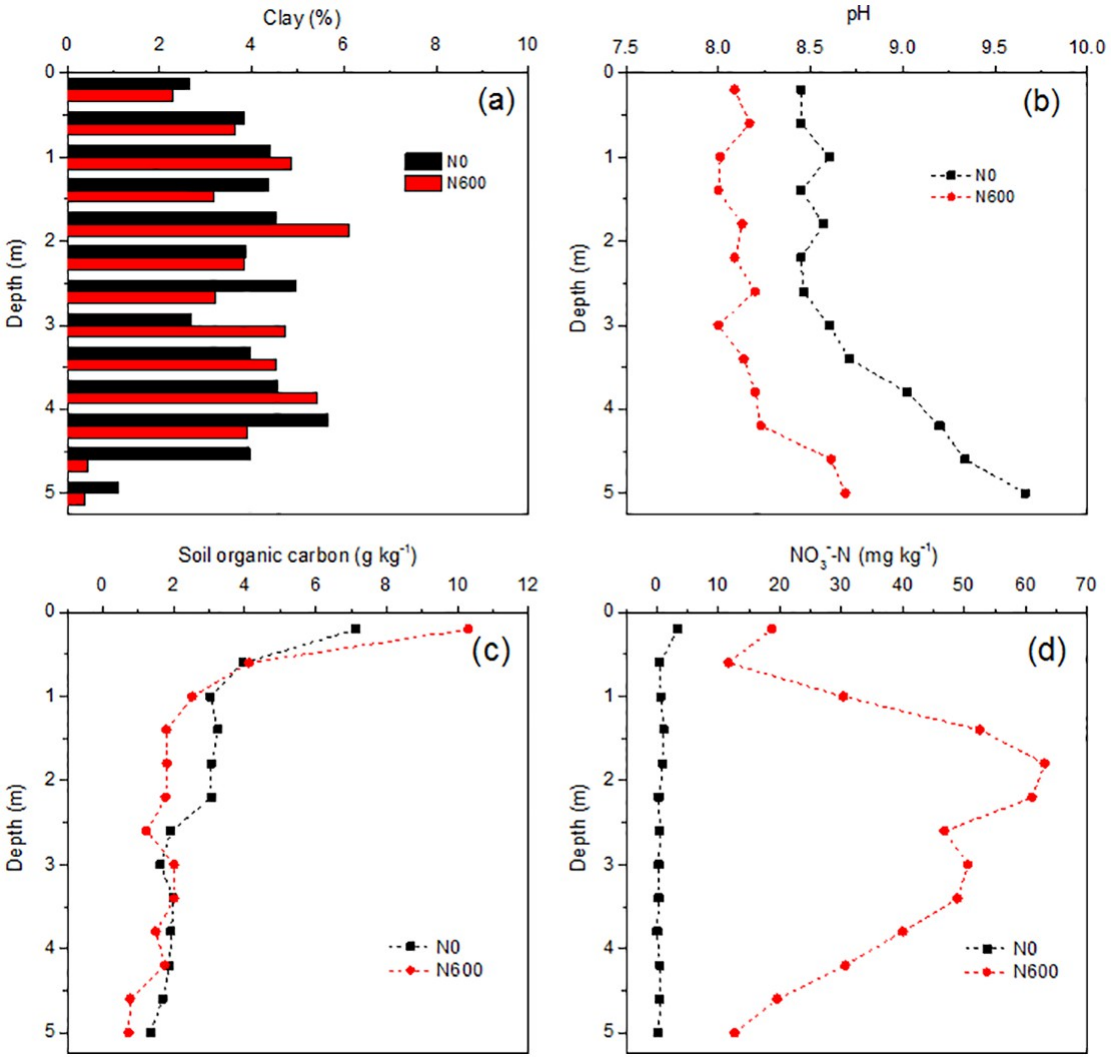
Soil chemical properties in different soil depths across the 0‒5.2 m soil profiles in the N0 and N600 treatments. Soil clay content (a), pH (b), soil organic carbon (c) and nitrate content (d) in different soil depths across the 0‒5.2 m soil profiles in the N0 and N600 treatments. N0 and N600 represent fertilizer N input rates of 0 and 600 kg N ha^-1^ year^-1^, respectively. Relative errors were less than 0.05 for all the measured parameters (n = 2).

The N_2_O/(N_2_O+N_2_) ratio in the 0‒5.2 m soil profile increased after 24 h of incubation with external N addition (N0+N) compared to that under the N0 treatment (Fig 1). Increase in the N_2_O/(N_2_O+N_2_) ratio was more remarkable at 0‒2.0 m depth. The ratio increased by 1.9% on average along the entire profile, indicating an immediate effect of N addition on the N_2_O/(N_2_O+N_2_) ratio. Previous studies have observed that N_2_O can be completely reduced to N_2_ at a low NO_3_^-^ addition (20 mg N kg^-1^), but is inhibited at a high NO_3_^−^-N addition (> 50 mg N kg^-1^) [13, 14]. The addition of 210 mg N kg^-1^ as NO_3_^−^-N in the present study, which was much higher than those applied in previous studies, similarly showed an effective immediate inhibition of N_2_O reductase activity. On an average, the N_2_O/(N_2_O+N_2_) ratio in the 0‒5.2 m soil profile under the N600+N treatment was 4.9% higher than that under the N0+N treatment (Fig 1), which showed the long-term effects of N addition on N_2_O reduction. If we regard the combined immediate and long-term effects as unit 1, then the results indicate that, apart from the immediate effect, 67.4% in the N_2_O/(N_2_O+N_2_) ratio resulted from the long-term effect of N addition. This was probably caused by a decrease in pH owing to the large amount of NO_3_^−^-N accumulation in the profile (Fig 2b and 2d).

Previous studies have shown that long-term N fertilization results in a decrease in soil pH and a consequent increase in the N_2_O/(N_2_O+N_2_) ratio [15, 16]. The pH across the profiles was about 0.5 units lower under the N600 treatment than that in the N0 treatment (Fig 2b), that is, the soil pH indeed decreased under the 15-year fertilizer N input. Pearson correlation analysis was performed to further test the correlation between soil properties and N_2_O ratio increase. The results showed that only the long-term effect of N addition on the N_2_O/(N_2_O+N_2_) ratio was significantly positively correlated with SOC content rather than clay and pH (Table 1). These results imply that more N_2_O is likely to be emitted in soils with excessive NO_3_^-^ and concurrent organic C enrichment. On the contrary, pH showed a negative correlation with the immediate or long-term effects of N input on N_2_O ratio increase (*r* = –0.28 and –0.35, respectively), but this was not statistically significant. This may be because even though the pH under N600 tended to decrease, the pH value was still in the alkaline range in this study due to the considerable calcium ion buffering capacity [17]. Nevertheless, N addition has significantly reduced pH by 0.26 pH-units on average globally (Tian and Niu, 2015). Under the trend of increasing atmospheric N deposition and continuous large N input for higher crop yields, soil acidification becomes more serious [18], which would thus have a greater impact on soil N_2_O emissions.

**Table 1:** Pearson correlation coefficients (r) between soil clay content, pH, and soil organic carbon (SOC) and the immediate and long-term effects of N addition on N_2_O emission ratio.

ΔN_2_O ratios†	Soil parameters		
	Clay	pH	SOC
**Immediate effects**	-0.113	-0.278	0.519
**Long-term effects**	-0.273	-0.350	0.911**

** Significant at *p* < 0.01 levels.

^†^ Immediate effects = R_[N0+N]_ − R_N0_; long-term effects = R_[N600+N]_ − R_[N0+N]_. N0+N and N600+N represent anaerobic incubation using the soil from zero and 600 kg N ha^-1^ year^-1^ fertilizer N treatment with external N addition, respectively. N0 was the same treatment, except for using the soil from the no-fertilizer N treatment.

## Conclusions

Using soil samples along a 0‒5.2 m soil profile from N0 and N600 fertilizer plots, we investigated the immediate and long-term effects of N addition on the N_2_O/(N_2_O+N_2_) ratio. High N fertilizer input (600 kg N ha^-1^ year^-1^) increased the N_2_O/(N_2_O+N_2_) emission ratio by 6.8%. Approximately 32.6% of the increase was due to the N_2_O reductase activity inhibition, and 67.4% of the increase was caused by comprehensive long-term effects, such as pH decrease or soil microbial community shift. There was a significant positive correlation between the long-term effects and SOC content. Future studies are needed to investigate soil acidification caused by N addition on the denitrifying community and end-product partition, especially in N-saturated and low-pH fields. Our results suggest that N addition-induced soil pH decrease should be included in models that predict biota communities and linkages to carbon and nitrogen cycling in terrestrial ecosystems under global environmental change scenarios, such as N deposition and soil acidification.

## Supporting information

S1 Data (XLSX)
